# The three steps method for uniportal video-assisted thoracoscopic right upper lobectomy

**DOI:** 10.1186/s13019-023-02129-0

**Published:** 2023-01-10

**Authors:** Caiyang Liu, Ran Ran, Lei Luo, Xiaoliang Li, Gaohua Liu, Hong Shao, Ji Li

**Affiliations:** 1 Department of Cardiothoracic Surgery, The First People’s Hospital of Neijiang, No. 1866, West Section of Hanan Avenue, Shizhong District, Neijiang, 641000 Sichuan China; 2 Breast Surgery Center of Sichuan Cancer Hospital, Chengdu, 610041 China

**Keywords:** Lung cancer, Video-assisted thoracoscopy, Right upper lobectomy

## Abstract

**Background:**

The uniportal video-assisted thoracoscopic right upper lobectomy (UVATRUL), as a common procedure for thoracic surgeons, is difficult to manipulate and has some inherent challenges. To solve both of problems, we summarized a series of techniques as the three steps method and investigated its feasibility on the patients of right upper lung cancer.

**Methods:**

Forty-eight patients with right upper lobe lung cancer who underwent the three steps method UVATRUL in our hospital from January 2020 to May 2022 were selected as the three steps method group. Forty-seven patients who underwent the traditional UVATRUL were selected as the traditional method group. The intraoperative condition and postoperative condition of the two groups were retrospectively analysed. Multiple linear regression analysis was carried out to analyze the relationship between positive results and surgical method.

**Results:**

All patients had successfully completed their surgeries. There was no significant difference between the two groups in respect of intraoperative blood loss, rate of conversion, day one thoracic drainage volume, chest tube indwelling time, incidence of postoperative complications, number of lymph node, and postoperative hospital stay (*P* > 0.05). Operative time of the three steps method group was significantly shorter than the traditional method group (*P* < 0.001), and number of reloads used was also significantly less than the traditional method group (*P* = 0.014). Multiple linear regression analysis showed that operative time (β = − 0.470, *P* < 0.001), and number of reloads (β = − 0.254, *P* = 0.007) correlated with surgical method.

**Conclusion:**

Compared with the traditional UVATRUL, the three steps method trims the surgery procedures, shortens the operative time, and reduces the use of reloads which makes it an effective procedure for UVATRUL.

**Supplementary Information:**

The online version contains supplementary material available at 10.1186/s13019-023-02129-0.

## Background

According to the “global cancer statistics”, lung cancer is one of the most common causes of cancer-related death worldwide, with increasing incidence and poor prognosis, remains a huge health threat to human beings [1]. Surgical management is the preferred treatment for most resectable non-small cell lung cancer (NSCLC) patients. Since video-assisted thoracoscopic surgery (VATS) was introduced in the early 1990s, and the first lobectomy was performed in 1992 [2], VATS represented a new trend in the development of minimally invasive thoracic surgery [3]. VATS lobectomy, usually performed through 2 to 4 incisions, allows multiple different angles of approach to the hilar structures and lymphatic tissues [4]. However, VATS lobectomy also can be accomplished with a single incision, with reduced access trauma, decreased postoperative pain, faster recovery, and improved patient satisfaction [5–7]. Uniportal VATS was initially reported by Rocco et al. [8], more than 10 years after that, it has made great progress because of the development of surgical technics and instruments. Nowadays, uniportal VATS becomes an increasingly popular approach to manage thoracic surgical diseases and some of the most complex thoracic procedures, such as sleeve lobectomy, anatomic segmentectomy and pulmonary artery reconstructions can also be performed [9, 10]. Uniportal video-assisted thoracoscopic right upper lobectomy (UVATRUL), as a common procedure for thoracic surgeons, has some inherent challenges [11]. Firstly, the horizontal fissure was always found hypoplastic which was once considered to be an indication for interim thoracotomy. However, Go’mez-Caro et al. [12] had completed VATS lobectomy successfully in patients with largely fused fissures using a "fissureless technique" to preserve pulmonary parenchyma. Based on this technique, Liu et al. [13] created a modified "single-direction procedure". Secondly, the upper lobe pulmonary vein is hard to be transected by stapler because of an inappropriate angle. Though making the uniportal incision in a lower intercostal space may solve this problem, we find it difficult to perform the dissection of level 2/4 lymph nodes in such a lower incision. To solve such problems in UVATRUL mentioned above, we summarized a series of techniques as the three steps method and investigated its feasibility on the patients of right upper lung cancer.

## Materials and methods

### Patients

Patients included were selected NSCLC patients who underwent UVATRUL by the same surgeon at the first people's hospital of Neijiang, China from January 2020 to May 2022, divided into a three steps method group and a traditional method group. The inclusion criteria were as follows: ① patients with right upper NSCLC and underwent UVATRUL; ② clinically early or advanced stage disease (T1-3N0-2M0), without invasion of surrounding tissues including the right middle lobe, the right lower lobe, and fissures; ③ maximum diameter of lymph nodes no more than 1 cm; ④ complete clinical data. Exclusion criteria were as follows: ① neoadjuvant therapy; ② intrathoracic extensive adhesions or pleural cavity atresia; ③ hilar lymph nodes calcifcation or fusion; ④ abnormal heart and pulmonary functions that could not suffer from surgery. Finally, the three steps method group comprised 48 patients and the traditional method group comprised 47 patients. We conformed that all examinations and treatments were performed in accordance with the relevant guidelines and regulations.

### Surgical methods

#### The three steps method

**Fig. 1:**
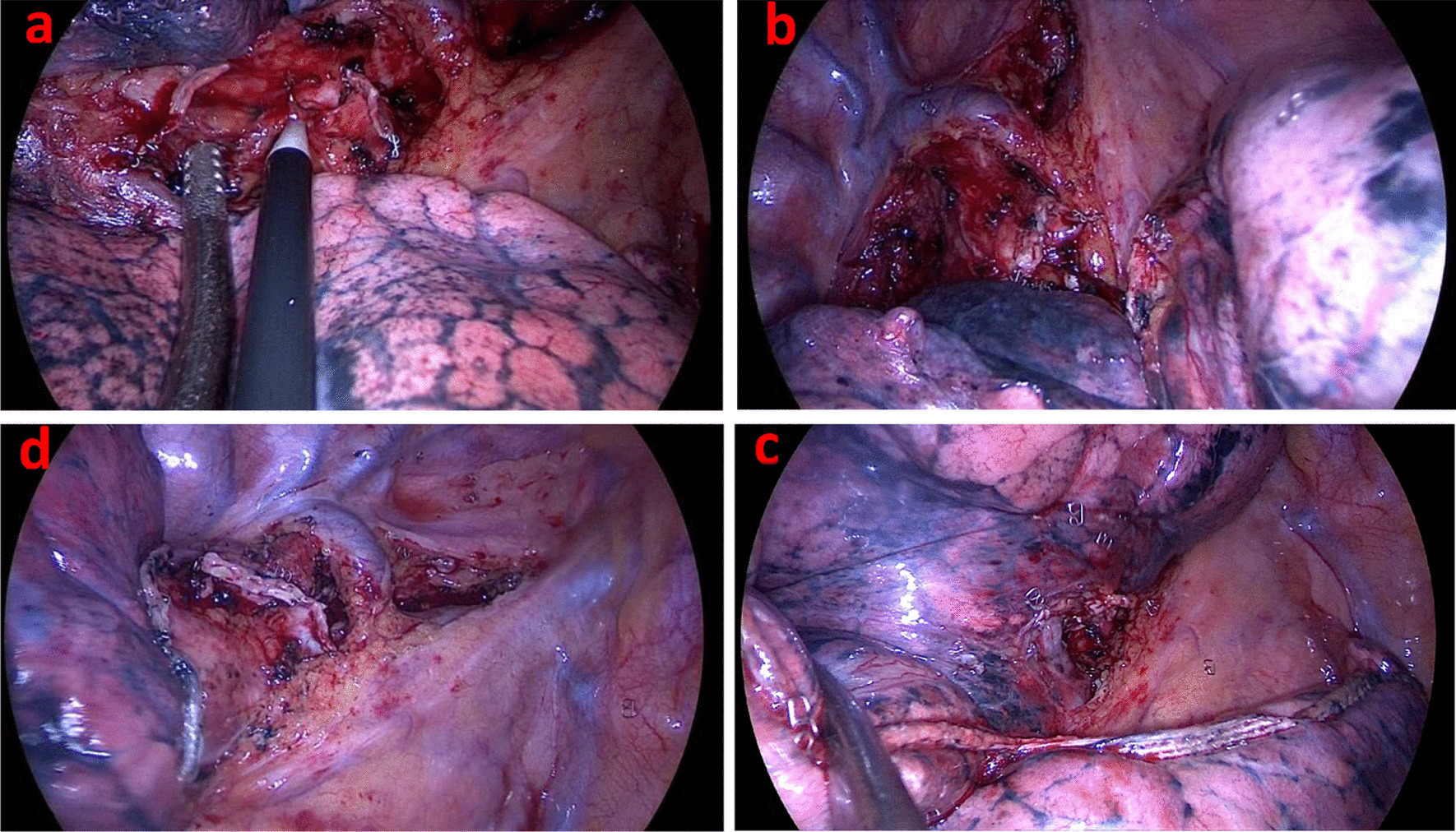
**a** Performing the dissection of level 10 and level 2/4 lymph nodes, and stapling the apico-anterior artery. **b** Performing the dissection of level 11 and level 7 lymph nodes, and stapling the upper lobe bronchus. **c** Stapling the horizontal fissure only but not the vein with the first reload. **d** All the rest structures could be stapled simultaneously

The uniportal incision located in the 5th intercostal space, anterior to latissimus dorsi and posterior to pectoralis major. After the devices entered the thoracic cavity, pulling the right upper lobe (RUL) backward to expose the anterior structures at the hilum. Step one: Cutting open the mediastinal pleura from horizontal fissure to right hilum pulmonis, identifying the middle lobe vein and the upper lobe vein separately, if necessary, dissecting both of them free. Performing the dissection of level 10 and level 2/4 lymph nodes, and stapling the apico-anterior artery (Fig. 1a). Step two: pulling the RUL forward, cutting open the mediastinal pleura from dorsal to cranial to expose the upper lobe bronchus, performing the dissection of level 11 and level 7 lymph nodes, and stapling the upper lobe bronchus (Fig. 1b). It was worth noting that the interlobar lymph nodes and hilar lymph nodes around the vessels should not be removed separately; rather, they should be dissociated to the distal end of the vessel and then removed en bloc with the RUL. Once such lymph nodes were dissociated, the posterior ascending artery was identified and the pulmonary artery could be dissected along its route to the peripheral side into the fissure to expose the right middle lobe and right lower lobe arteries. Step three: Since the posterior half of the RUL was almost empty, all the rest structures of the RUL including the posterior ascending artery, the fissures, and the RUL vein could be stapled from the anterior to the posterior side along the fissures simultaneously (Fig. 1c). At this point, taking care to protect the middle and lower arteries because theirs supply might be jeopardized by staplers. In addition, to avoid the possibility of the vein tear, it was better to staple the horizontal fissure only with the first reload and staple the vein alone with another reload. Finally, removing the lung tissues with a specimen bag and performing the dissection of the rest lymph nodes (Fig. 1d).

#### The traditional method

Did the same uniportal incision as the three steps method. The order of RUL hilum anatomy for lobectomy was the main different between these two methods. Here was the order of traditional method: the upper lobe pulmonary vein → the apico-anterior artery → the posterior ascending artery → the upper lobe bronchus → the fissures. Surgeons might change this order according to the actual situations. Then removing the lung tissues and performing dissection of the mediastinal lymph nodes. This method had been described in detail in previous literature [14, 15].

### Statistical analysis

If normally distributed, continuous variables were expressed as mean ± standard deviation and compared by the student’s t-test; if not normally distributed, variables were expressed as median (interquartile range) and compared by the Mann–Whitney U test. Categorical variables were expressed as counts and percentages, and were compared with chi-square test or Fisher’s exact test. We conducted linear regression analysis to examine factors that could be related to positive results and possible influence factors were subjected to multiple linear regression analysis. The difference was statistically significant at *P* < 0.05. SPSS 22.0 (IBM Corp., Armonk, NY, USA) software was used for the statistical analysis.

## Results

### Patients characteristics

Of the 95 NSCLC patients, there were 48 men and 47 women. The age, smoking history, body mass index, forced expiratory volume in 1 s, location of primary, mean tumor diameter, stage, and pathological type between the two groups were not significantly different (*P *˃ 0.05). The clinical characteristics of the two groups were shown in Table 1.

**Table 1:** Comparison of clinical characteristics between the two groups

Clinical characteristics	Groups		*P* value
	Three steps method (n = 48)	Traditional method (n = 47)	
Age (years)	60.19 ± 10.43	61.57 ± 11.31	0.536
Gender			0.473
Male	26(54.17%)	22(46.81%)	
Female	22(45.83%)	25(53.19%)	
Smoking			0.877
Yes	15(31.25%)	14(29.79%)	
No	33(68.75%)	33(70.21%)	
BMI	22.81 ± 2.92	23.22 ± 2.89	0.497
FEV1	2.26 ± 0.41	2.32 ± 0.46	0.449
Location of primary			0.777
Peripheral	42(87.50%)	42(89.36%)	
Central	6(12.50%)	5(10.64%)	
Mean tumor diameter (cm)	2.42 ± 0.92	2.51 ± 0.98	0.662
pTNM stage			0.947
I	39(81.25%)	37(78.72%)	
II	7(14.58%)	8(17.02%)	
IIIA	2(4.17%)	2(4.26%)	
Pathological type			0.739
Adenocarcinoma	34(70.83%)	36(76.60%)	
Squamous cell carcinoma	9(18.75%)	8(17.02%)	
Others	5(10.42%)	3(6.38%)	

*BMI* body mass index, *FEV1* forced expiratory volume in 1 s

### Surgical outcomes

All patients had successfully completed their surgeries. There was no perioperative death. All patients have a negative surgical margin. The blood loss, probability of conversion to multi-port or open surgery, day 1, chest tube drainage, duration with tube, complications, number of lymph node, and length of hospital stays between the two groups were not significantly different (*P* ˃ 0.05), whereas the operative time in the three steps method group was significantly shorter than the traditional method group (*P *˂ 0.001), and the reloads used in the three steps method group was significantly less than the traditional method group (*P* = 0.014). The surgical outcomes of the two groups were shown in Table 2. Multiple linear regression analysis showed that operative time (β = − 0.470, *P* < 0.001), and number of reloads (β = − 0.254, *P* = 0.007) correlated with surgical method (Additional file 1).

**Table 2:** Surgical outcomes between the two groups

Surgical outcomes	Groups		*P* value
	Three steps method (n = 48)	Traditional method (n = 47)	
Thoracoscopic procedure (min)	110.04 ± 33.01	146.64 ± 34.46	** < 0.001**
Blood loss (ml)	136.46 ± 103.52	134.04 ± 88.53	0.903
Conversion to multi-port or open surgery	1(2.08%)	2(4.26%)	0.545
Day 1, chest tube drainage (ml)	173.23 ± 71.34	157.87 ± 70.25	0.293
Duration with tube (day)	4.83 ± 1.71	5.28 ± 2.08	0.259
Complications	12(25.00%)	13(27.66%)	0.769
Air leakage	6(12.50%)	8(17.02%)	–
Hemorrhage	0(0.00%)	0(0.00%)	–
Pulmonary infection	7(14.58%)	6(12.77%)	–
Length of hospital stays (day)	6.92 ± 1.76	7.72 ± 2.20	0.051
Number of reloads	6.65 ± 0.84	7.11 ± 0.96	**0.014**
Number of lymph node	11.83 ± 2.85	12.53 ± 2.78	0.229
Perioperative mortality (%)	0	0	–

## Discussion

More and more small nodules such as groundglass nodule were detected because of the prevalence of low-dose computer tomography and treated by surgery [16]. About ten years ago, the long-term outcomes of VATS for early stage lung cancer had been proved to be similar with thoracotomy [17, 18]. Henceforth, thoracic surgeons have tried their best to minimize the invasion of VATS in order to improve the quality of life postoperatively, in such a case, uniportal VATS was applied in lobectomy for lung cancer, even for advanced stages cases [19]. However uniportal VATS requires higher level of acquainted anatomical knowledge, special surgical strategy and skill and experience for VATS trouble-shooting. UVATRUL was a common operation for thoracic surgeons. Procedure of the traditional method including division of the fissures, ligation of the RUL vein, apico-anterior artery, and posterior ascending artery, and stapling of the bronchus [20]. Although different surgeons might obey different orders, generally speaking, the procedure of traditional method was kind of cumbersome. Uniportal VATS was originally difficult to manipulate, let alone the inherent challenges of UVATRUL. Surgeons with less experience might find the traditional method of UVATRUL hard to perform and in some specific situations surgeons have to make a conversion to multi-port or open surgery, for example largely fused fissures and small thoracic cavity. So we thought that a trimmed surgery procedure was needed. We summarized a series of techniques as the three steps method which might serve as an alternative surgery procedure.

Compared with the traditional method, there are several advantages of the three steps method. Firstly, we perform the dissection of lymph nodes before we staple the upper lobe bronchus and the apico-anterior artery because it enables better exposure of the level 7 and level 2/4 lymph nodes and avoids frequent turn-over of the lung lobes during the lobectomy and lymph node dissection. Secondly, dealing with the the apico-anterior artery first thus effectively avoids its’ unexpected damage in traditional method when surgeons dissociate and transect the upper lobe pulmonary vein. Finally, we staple all the rest structures of the RUL except the bronchus and apico-anterior artery simultaneously, theoretically shortens the operative time, reduces the use of reloads, and decreases the probability of bleeding and air leakage. However we are conscious that there are still some problems to be solved. For example, it is important to identify the middle lobe vein and the upper lobe vein separately before we perform the last step, because there is chance of accidental endo-stapling of the middle lobe vein which may lead to venous infarction in the middle lobe. Besides, the three steps method should not be performed once the tumor invade the fissures. What’s more, some N1 nodes are deemed to be resected simultaneously in the last step without adequate sampling or dissection, which may compromise the oncological principle in lung cancer surgery.

Except the traditional method there were still other methods reported. Ten years ago, Liu et al. [13] reported the single-direction thoracoscopic lobectomy and they advocated performing lobectomy progressively in a single direction from superficial to deep structures. It overcomes the difficulty in manipulation of incomplete lung fissures and makes the procedure of lobectomy clear. But this method is more suitable for multiple-port VATS right upper lobectomy, because the inappropriate angle under uniport makes the RUL vein hard to be transected by stapler. In addition, changes in anatomical order and position may lead to misoperation within surgeons with less experience. Four years ago, Zhang et al. [20] introduced a bronchus-first and simultaneous vessel stapling method for VATS right upper lobectomy. They stapled bronchus and vessels respectively to avoid the bronchi-vascular fistulas and vessel tears. But they had to face the challenge of hypoplastic fissures first before they made the next move.

In the entire group, there were no perioperative death and severe complications. The average operation time was 110.04 min, which appears shorter than Liu’s single-direction method and the same as Zhang’s bronchus-first and simultaneous vessel stapling method. The surgical outcomes were favorable. However there were several limitations of this study should be addressed: ①potential selection bias and information bias due to this article being a single-center retrospective study; ② patients included within two years, there were no long-term outcomes obtained; ③the sample size was relatively small.

In summary, the three steps method trims the surgery procedures, shortens operative time, and reduces the use of reloads which makes it an effective procedure for UVATRUL.

## Supplementary Information

**Additional file 1.** Multiple linear regression analysis showed that operative time and number of reloads correlated with surgical method.

## Data Availability

All data generated or analysed during this study are included in this published article.

## Abbreviations

VATS: Video-assisted thoracoscopic surgery
UVATRUL: Uniportal video-assisted thoracoscopic right upper lobectomy
NSCLC: Non small cell lung cancer
RUL: Right upper lobe

## References

[1] Jemal A, Bray F, Center MM (2011). Global cancer statistics. CA Cancer J Clin.

[2] Lewis RJ (1993). The role of video-assisted thoracic surgery for carcinoma of the lung: wedge resection to lobectomy by simultaneous individual stapling. Ann Thorac Surg.

[3] Swanson SJ, Herndon JE, D'Amico TA (2007). Video-assisted thoracic surgery lobectomy: report of CALGB 39802—a prospective, multi-institution feasibility study. J Clin Oncol.

[4] Rocco G (2013). VATS and Uniportal VATS: a glimpse into the future. J Thorac Dis.

[5] McElnay PJ, Molyneux M, Krishnadas R (2015). Pain and recovery are comparable after either uniportal or multiport video-assisted thoracoscopic lobectomy: an observation study. Eur J Cardiothorac Surg.

[6] Jutley RS, Khalil MW, Rocco G (2005). Uniportal vs standard three-port VATS technique for spontaneous pneumothorax: comparison of post-operative pain and residual paraesthesia. Eur J Cardiothorac Surg.

[7] Tamura M, Shimizu Y, Hashizume Y (2013). Pain following thoracoscopic surgery: retrospective analysis between single-incision and three-port video-assisted thoracoscopic surgery. J Cardiothorac Surg.

[8] Rocco G, Martin-Ucar A, Passera E (2004). Uniportal VATS wedge pulmonary resections. Ann Thorac Surg.

[9] Gonzalez-Rivas D, Mendez L, Delgado M (2013). Uniportal video-assisted thoracoscopic anatomic segmentectomy. J Thorac Dis.

[10] Gonzalez-Rivas D, Delgado M, Fieira E (2014). Double sleeve uniportal video-assisted thoracoscopic lobectomy for non-small cell lung cancer. Ann Cardiothorac Surg.

[11] McKenna RJ, Houck W, Fuller CB (2006). Video-assisted thoracic surgery lobectomy: experience with 1,100 cases. Ann Thorac Surg.

[12] Go´mez-Caro A, Calvo MJ, Lanzas JT (2007). The approach of fused fissures with fissureless technique decreases the incidence of persistent air leak after lobectomy. Eur J Cardiothorac Surg.

[13] Liu L, Che G, Qiang P (2009). A new concept of endoscopic lung cancer resection: Single-direction thoracoscopic lobectomy. Surg Oncol.

[14] He CJ (2018). Application and experience of single endoscopic linear cutter stapler through uniportal video-assisted thoracic surgery for lung cancer in right upper lobe. Oncol Progress.

[15] He J, Ma D, Li S (2017). Uniportal video-assisted thoracoscopic right upper lobectomy and systemic mediastinal lymph nodes dissection. J Thorac Dis.

[16] Henschke CI, Yip R, Smith JP (2016). CT screening for lung cancer: part-solid nodules in baseline and annual repeat rounds. AJR Am J Roentgenol.

[17] Paul S, Isaacs AJ, Treasure T (2014). Long term survival with thoracoscopic versus open lobectomy: propensity matched comparative analysis using SEER-Medicare database. BMJ.

[18] Lee PC, Nasar A, Port JL (2013). Long-term survival after lobectomy for non-small cell lung cancer by video-assisted thoracic surgery versus thoracotomy. Ann Thorac Surg.

[19] Gonzalez-Rivas D, Fieira E, Delgado M (2014). Is uniportal thoracoscopic surgery a feasible approach for advanced stages of non-small cell lung cancer?. J Thorac Dis.

[20] Zhang L, Hao X (2018). Video-assisted thoracic surgical right upper lobectomy with bronchus-first and simultaneous vessel stapling technique. Thorac Cardiovasc Surg.

