# Supplementary material for: The three steps method for uniportal video-assisted thoracoscopic right upper lobectomy
Source: J Cardiothorac Surg. 2023 Jan 10;18:12. doi: 10.1186/s13019-023-02129-0 (PMC9830912; doi:10.1186/s13019-023-02129-0)
Supplement: Supplementary file 1 — Additional file 1. Multiple linear regression analysis showed that operative time and number of reloads correlated with surgical method. [file 13019_2023_2129_MOESM1_ESM.docx]

| Model | | Unstandardized Coefficients | | Standardized Coefficients | t | Sig. | 95.0% Confidence Interval for B | |
| --- | --- | --- | --- | --- | --- | --- | --- | --- |
|  |  | B | Std. Error | Beta |  |  | Lower Bound | Upper Bound |
| 1 | (Constant) | 8.457 | .731 |  | 11.576 | .000 | 7.005 | 9.909 |
|  | Gender | .321 | .193 | .174 | 1.665 | .100 | -.062 | .704 |
|  | BMI | -.080 | .030 | -.249 | -2.692 | .009 | -.138 | -.021 |
|  | Smoking | -.535 | .211 | -.268 | -2.530 | .013 | -.955 | -.115 |
|  | pTNM stage | .473 | .162 | .266 | 2.914 | .005 | .150 | .796 |
|  | Pathological type | -.294 | .139 | -.201 | -2.119 | .037 | -.571 | -.018 |
|  | Method | -.467 | .168 | -.254 | -2.775 | .007 | -.802 | -.133 |
| a. Dependent Variable: **Number of reloads** | | | | | | | | |

| Model | | Unstandardized Coefficients | | Standardized Coefficients | t | Sig. | 95.0% Confidence Interval for B | |
| --- | --- | --- | --- | --- | --- | --- | --- | --- |
|  |  | B | Std. Error | Beta |  |  | Lower Bound | Upper Bound |
| 1 | (Constant) | 124.530 | 30.500 |  | 4.083 | .000 | 63.937 | 185.124 |
|  | BMI | -1.782 | 1.101 | -.135 | -1.618 | .109 | -3.970 | .406 |
|  | FEV1(L) | 12.974 | 7.396 | .147 | 1.754 | .083 | -1.719 | 27.667 |
|  | Pathological type | 26.548 | 6.075 | .361 | 4.370 | .000 | 14.480 | 38.617 |
|  | Method | -35.749 | 6.304 | -.470 | -5.671 | .000 | -48.272 | -23.225 |
| a. Dependent Variable: **Operative time (min)** | | | | | | | | |
